# Vascular Endothelial Cells: Heterogeneity and Targeting Approaches

**DOI:** 10.3390/cells10102712

**Published:** 2021-10-10

**Authors:** Jan K. Hennigs, Christiane Matuszcak, Martin Trepel, Jakob Körbelin

**Affiliations:** 1 ENDomics Lab, Department of Oncology, Hematology and Bone Marrow Transplantation with Section Pneumology, University Medical Center Hamburg-Eppendorf, 20246 Hamburg, Germany; chrisitiane.matuszcak@gmail.com; 2 Department of Hematology and Medical Oncology, University Medical Center Augsburg, 86156 Augsburg, Germany; martin.trepel@uk-augsburg.de

**Keywords:** endothelial cell, vascular endothelial cell, endothelium, vasculature, vascular targeting, endothelial heterogeneity

## Abstract

Forming the inner layer of the vascular system, endothelial cells (ECs) facilitate a multitude of crucial physiological processes throughout the body. Vascular ECs enable the vessel wall passage of nutrients and diffusion of oxygen from the blood into adjacent cellular structures. ECs regulate vascular tone and blood coagulation as well as adhesion and transmigration of circulating cells. The multitude of EC functions is reflected by tremendous cellular diversity. Vascular ECs can form extremely tight barriers, thereby restricting the passage of xenobiotics or immune cell invasion, whereas, in other organ systems, the endothelial layer is fenestrated (e.g., glomeruli in the kidney), or discontinuous (e.g., liver sinusoids) and less dense to allow for rapid molecular exchange. ECs not only differ between organs or vascular systems, they also change along the vascular tree and specialized subpopulations of ECs can be found within the capillaries of a single organ. Molecular tools that enable selective vascular targeting are helpful to experimentally dissect the role of distinct EC populations, to improve molecular imaging and pave the way for novel treatment options for vascular diseases. This review provides an overview of endothelial diversity and highlights the most successful methods for selective targeting of distinct EC subpopulations.

## 1. Overview

The vascular endothelium is a heterogenous monolayer of highly specialized cells, the endothelial cells (ECs), that face the luminal side of all blood vessels and represent the first barrier for all molecules, cells or pathogens circulating in the bloodstream [1]. Their cumulative surface area is estimated to be approximately 1000 m^2^. Therefore, the vascular endothelium can be considered the largest organ in the human body [2]. Indeed, this unique cell layer acts as an endocrine organ and constitutes a selectively permeable barrier between extra- and intravascular compartments by controlling the exchange of compounds between the bloodstream and the surrounding tissue in an organ-specific manner [3,4,5,6,7]. The luminal surface of all vascular endothelial cells is covered by the endothelial glycocalyx, which comprises membrane-bound, negatively charged proteoglycans, glycolipids and glycosaminoglycans, thereby contributing to mechanotransduction, cell signaling and adhesion of blood cells and pathogens [1]. Some of the targeting agents discussed in this review utilize components of the endothelial glycocalyx as primary attachment receptors. Disruption or dysfunction of the glycocalyx has been associated with diseases such as diabetes, chronic kidney disease, inflammatory condition, sepsis, hypernatremia, hypervolemia and ischemia/reperfusion injury [8]. Changes in the glycocalyx have also been linked to the treatment response in sepsis [9]. Although all blood vessels share common functions, such as oxygen and metabolite transport, not all blood vessels and especially not all ECs are equal. Even the most common EC markers, such as CD31 and the von Willebrand Factor (vWF), are expressed in a heterogeneous pattern along different EC populations [10]. Indeed, blood vessels of different compartments of the organism express specific markers. A recently published comprehensive single-cell transcriptome atlas of murine ECs reveals the vast diversity of vascular EC on the transcriptional level alone [11]. Vascular ECs adapt to their specific niche in the context of the individual organs by highly specialized functions (e.g., blood–brain barrier, glomeruli in the kidney, or sinusoids in the liver) [12]. ECs differ morphologically, physiologically and phenotypically along the vascular tree, between arteries and veins, arterioles and venules, even within capillaries of the same vascular beds [13] and between different organs within the body [14,15]. These differences manifest in variable permeability, responsiveness and biosynthesis [6]. Age and sex also influence EC gene expression signatures [16] and, importantly, EC gene expression can differ significantly between species, even when comparing identical vascular compartments [17]. ECs developed in ancestral vertebrates, approx. 510–540 million years ago, followed by further specialization [18]. Key components of the vascular system, such as the endothelial blood–brain barrier (BBB), presumably arose several times during evolution [19,20]. Potential inter-species differences, even between mammals [17] are particularly important, as many of the studies discussed in this review have been conducted with mice or other rodents and the respective findings do not necessarily translate to human cells.

As a multifunctional boundary layer, the endothelium is responsible for receiving and translating signals from the blood [21]. All changes in the circulating blood are perceived by the endothelium which then mediates signal transduction to the other layers of the vascular wall. Such changes include mechanical stress (elongation and wall shear stress), as well as changes in the concentration of metabolic factors. The expression of distinct receptor molecules enables the response to growth factors, such as the vascular endothelial growth factor (VEGF) or basic fibroblast growth factor (bFGF), hormones and cytokines, such as interleukins or to bacterial toxins. Vascular ECs convey the balance between coagulation and fibrinolysis and play a major role in the regulation of immune response and inflammation [6]. The endothelium is involved in the regulation of the vascular tone by secreting vasodilatory factors, such as nitric oxide (NO) and prostacyclin (PGI2), or vasoconstrictive molecules, such as angiotensin or endothelin-1 and -2 [22,23,24,25,26]. Vascular ECs are also important in regulating growth and homeostasis of the adjacent layer of smooth muscle cells by secreting various paracrine factors [27].

The process of generating new blood vessels from pre-existing ones—angiogenesis—is largely initiated by growth factor receptors expressed on the surface of vascular ECs, mainly by vascular endothelial growth factor receptors (VEGFRs) [6]. Angiogenesis regulates the adaptation or formation of new vessels in the organism in order to maintain the supply of oxygen and nutrients. The endothelial VEGFR-2 expression is highly upregulated in cancer and various ischemic diseases [28] and therefore, it enables preferential targeting of tumor ECs, as discussed further below. As vascular ECs also control adhesion and transmigration of circulating blood and tumor cells via the expression of surface receptors, they are pivotal in mediating inflammatory and immune responses, as well as being important for tumor evasion and metastasis [6,29,30,31]. At the vascular level, inflammatory events include, among other processes, the dilation of arterioles, capillaries and venules, leading to increased permeability and increased blood flow, as well as trans-endothelial migration of leukocytes into the tissue [32]. In the event of an infection, the activation of the endothelium leads to excessive expression of inflammatory proteins such as adhesion molecules on the cell surface, thus enabling leukocytes to bind to the endothelium and to transmigrate into the affected tissue [33].

Further, the vascular endothelium is involved in coagulation. In this context, vWF plays an important role. As a carrier and protective protein of the coagulation factor VIII, vWF is found in endothelial storage granules, so-called Weibel–Palade bodies [34]. In the event of a vascular injury, vWF is released and binds to the damaged vessel wall, as well as to the platelets. This confers platelet aggregation within the damaged area, which results in the formation of blood clots covering the damaged area, thus preventing further leakage of blood into the surrounding tissue.

## 2. Physiological Endothelial Heterogeneity

### 2.1. Endothelial Heterogeneity along the Vascular Tree

Arterial and venous blood vessels are morphologically and genetically distinct due to different functions in the body [35,36]. Arteries and veins are composed of different layers. The luminal intima is composed of ECs and their glycocalyx, the media consists of smooth muscle cells and elastic connective tissue, whereas the abluminal adventitia is composed of connective tissue and collagen-producing fibroblasts. In humans, arteries and veins range from approx. 0.1 mm to 2.5 cm in diameter [37]. Arterioles and venules have diameters between 8 µm and 0.1 mm. In principle, their walls are composed of the same three layers as the larger vessels although media and adventitia are much thinner and less pronounced. Post-capillary venules below 30 µm in diameter completely lack media and adventitia and only consist of ECs and a basement membrane [37]. Precapillary arterioles are highly important in regulating vascular tone and contribute most to the total vascular resistance [38], whereas postcapillary venules are the main site of leukocyte trafficking [36]. Capillaries the smallest vessels of the body (>8 µm diameter). Most capillaries only consist of a single layer of ECs and a basement membrane [37], while others are supported by abluminal cells such as podocytes in the kidney glomeruli [39], or pericytes and astrocytes in blood-brain barrier capillaries [40]. In fact, the majority of vascular ECs is attributed to the extensive capillary network. With their thin walls and their enormous surface area, capillaries are responsible for gas exchange and metabolite transfer between blood and the individual organs in the body [5]. As blood vessels show large variability in size, morphology and function, there are distinct EC subpopulations along the vascular tree. Selective gene expression enables the discrimination between ECs of large vessels and the microvasculature. In a cell culture-based microarray Chi et al. identified over 500 genes specifically expressed in ECs from large vessels and more than 2500 genes distinctively expressed in microvascular ECs [41]. The majority of the genes specific for large vessel ECs are involved in biosynthesis and extracellular matrix remodeling. Interestingly, expression levels of common EC markers, such as vWF and VCAM1 appear to be directly correlated with vessel diameter [42]. In microvascular ECs, expression of genes encoding basement membrane proteins is predominant [42]. Vessel size is not the only factor contributing to the expression profile of ECs. Function and localization within the vascular tree are additional important factors, as arterial and venous ECs can be distinguished according to their gene expression profiles. Examples of markers enabling the distinction between arterial and venous ECs include Neuropilin 1 [43], Notch1, -4 and -5 [44,45] and Alk1 [46] for arterial ECs and Neuropilin 2 [47], Lefty-1 & 2 [41] as well as Endomucin [48,49] for venous ECs. Many other genes have been reported to be selectively expressed either in arterial or in venous ECs, which has been comprehensively reviewed before [36]. The ECs of the lymphatic system, not being focus of the current review, seem to derive from venous structures during embryogenesis [50,51,52,53,54]; yet, they can clearly be distinguished from venous vascular ECs (and from vascular ECs in general) by typical lymphatic markers such as LYVE-1 or Prox-1 as well as by high expression of Synaptogyrin 3 and various genes encoding proteins of the soluble N-ethylmaleimide-sensitive factor attachment protein receptor (SNARE) family while they lack VCAM and N-cadherin [55].

Being in direct contact with the blood, vascular ECs are exposed to different biomechanical and biochemical parameters, many of which can be sensed and further contribute to vascular heterogeneity. The endothelial response to these stimuli results in rapid, short-term or long-term adaptation processes of the vascular system. Hemodynamic forces that act on the vessel wall through blood flow are of particular importance for the control of various adaptive processes of the vascular system [56,57,58,59]. A considerable number of genes (~350) have been described to be differentially expressed upon changes of hemodynamic forces (in particular by stretching forces and shear stress) [60,61,62,63]. Recently, a KLF4/SWI/SNF complex has been identified as a master regulator of chromatin remodeling upon endothelial shear stress [64]. Therefore, adaptive gene expression enables ECs to adjust the vascular system to hemodynamic changes by regulating angiogenesis, vascular tone, migration and differentiation [60]. However, the biophysical and biochemical properties of the blood flow do not seem to be the only regulators of the vascular heterogeneity [36]. The expression of the specific marker Ephrin-B2 in developing arterial vascular structures and that of the EphB4 receptor in venous vascular structures occur very early in vascular development, even before blood circulation is established. This indicates that the molecular determination into an arterial or venous subtype is subject to early genetic control [36,65,66,67].

### 2.2. Endothelial Heterogeneity within the Capillary Network

**Figure 1:**
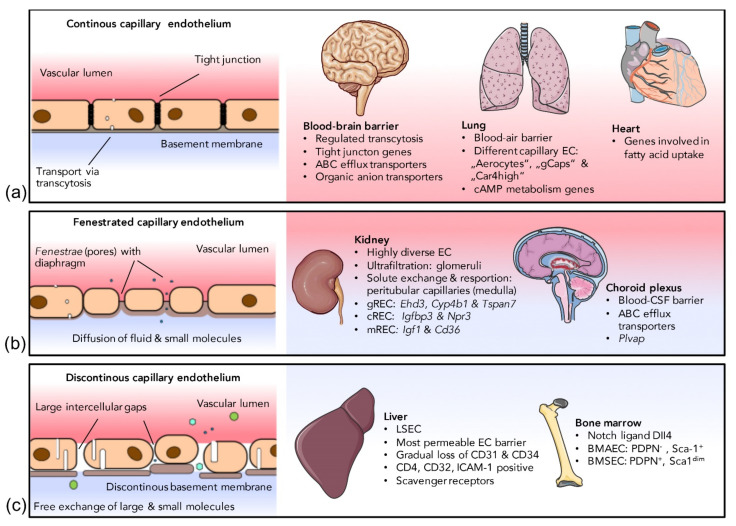
Three main types of capillary EC. (**a**) A tight continuous endothelium with a continuous basement membrane can be found in the capillaries of organs such as the brain, the lung and the heart. Molecules can pass the continuous endothelium by tightly regulated transcytosis. (**b**) The endothelium of the kidney and the choroid plexus is fenestrated and allows for diffusion of fluids and small molecules. (**c**) The capillary endothelium of liver and bone marrow is discontinuous with intercellular gaps and a discontinuous basement membrane, enabling free exchange of molecules. This figure contains artwork components of Servier Medical Art.

In addition to phenotypic differences along the vascular tree, the endothelium shows a strong heterogeneity due to adaption to local requirements in the specific vascular niche, which is reflected by classification into three main groups, namely, continuous, fenestrated and discontinuous endothelium [68]. Capillaries can be attributed to at least one of the three different endothelial classes depending on the organ they are found in. A schematic overview is presented in Figure 1. Although the highest diversity of capillary ECs can be found on the inter-organ level, many organs, such as the kidney, show an astonishing intra-organ EC diversity [69]. The organ-specific differentiation into EC subtypes is induced by the response of endothelial promoters and regulatory elements to specific transcription factors which, in turn, are influenced by the EC microenvironment. It has been shown, in transgenic mice, that a fragment of the 5′ non-coding region of the vWF gene drives reporter gene expression in the ECs of the brain, heart and skeletal muscles but not in the ECs of other organs. Further, vWF expression driven by the same promoter fragment was specifically induced upon co-culture of cardiac microvascular ECs with cardiomyocytes, whereas co-culture with hepatocytes or fibroblast did not induce vWF expression [70]. Regulatory elements necessary for activating vWF expression in other organs, such as the the lung, have been identified later [71,72]. A similar example is an isolated 1 kb fragment of the Flt-1 promoter which was shown to induce EC expression in all vascular beds except for the liver [73]. Although the full-length promoter sequences in the above-mentioned cases allow for gene expression in virtually all ECs, these studies prove that microenvironment-dependent transcription factors interact with respective regulatory elements on pan-endothelial promoters in an organ-specific manner. Other promoters or promoter elements seem to be induced only in distinct EC subpopulations [74]. Brain EC-specific expression of Cre recombinase driven by the *Slco1c1* promoter, for example, has successfully been employed to generate a mouse model with specific gene knockout in cerebral ECs [75]. Of note, there is considerable interspecies variability in gene expression of EC subtype-specific genes. The selective gene expression of *Slco1c1* in murine brain ECs, for example, is not seen in humans [76].

#### 2.2.1. Continuous Endothelium

The cells of the continuous endothelium are connected by tight junctions. The transcytosis rate is low and well-regulated and the EC monolayer is attached to a continuous basement membrane [14]. Barrier-forming continuous endothelium can be found in most arteries and veins, skeletal muscle, heart, adipose tissue, lung, skin and the central nervous system.

**Blood-brain barrier:** The BBB is the most restrictive and probably best-studied EC barrier in mammals, tightly regulating the brain’s fragile micromilieu [77]. Many severe neurovascular diseases such as multiple sclerosis [78], ischemic stroke [79] or Alzheimer’s disease [80], are associated with BBB dysfunction. Although the BBB is formed by an interplay of ECs, pericytes and astrocyte endfeet, the barrier properties are mostly determined by the endothelial layer [77]. Transcytosis in brain ECs is tightly regulated and a transmembrane protein encoded by the *Mfsd2a* gene, which is specifically expressed in CNS EC, has been shown to be responsible for suppressing transcytosis [81]. Despite forming intact tight junctions, *Mfsd2a* knockout mice develop a leaky BBB, attesting to the importance of controlled transcytosis for a functional BBB [81]. In an analysis of the mouse BBB transcriptome, Daneman et al. found a specific gene expression signature for brain ECs compared to parenchymal non-ECs from the brain, as well as ECs from peripheral organs [82]. Many of these BBB-specific genes encode selective transmembrane transporters. The most strongly enriched transcripts in the BBB include *Itih5*, encoding an extracellular matrix protein, *Apcdd1*, encoding a protein involved in coordinating vascular remodeling [83], a set of efflux transporter genes such as *Abcb1a*, as well as genes encoding multiple organic anion transporters such as *Slco1c1* [82], which had already been demonstrated to be expressed in cerebral ECs by another group [84]. The high expression rate of transmembrane transporters in cerebral ECs has recently been confirmed in another comprehensive EC transcriptome analysis [11]. Due to their unique gene expression pattern, ECs of the CNS can selectively be targeted by different approaches, some of which are discussed further below in the fourth section of this review.

**Blood-retina barrier:** The blood–retina barrier (BRB) consists of two layers; the outer layer is formed by tight junctions between retinal pigment epithelial cells, while the inner layer (iBRB) is formed by a tight continuous endothelium at the interplay with pericytes, Müller cells and astrocyte endfeet, very similar to the BBB discussed above. Diabetic retinopathy, one of the most frequent retinopathies, is directly linked to a dysfunctional iBRB [85]. The iBRB and the BBB share a set of characteristic features, namely tight junctions and a multitude of transporters allowing for directed trans-endothelial movement of solutes in order to compensate for reduced vesicular transport. Although showing many similarities, such as strong expression of tight junction and solute carrier genes, a recent comparative transcriptome analysis revealed some differences between microvessels of the iBRB and the BBB [86]. The expression level of the tight junction-associated genes *Cx43*, *Cx40* and *Ocln* was shown to be significantly lower in the iBRB than in the BBB, potentially contributing to a higher susceptibility to systemic challenges [86]. Indeed, *Cx43* has been shown to be crucial for maintaining an intact iBRB and to be declined in hyperglycemia [87,88]. Similarly, the insulin-sensitive glucose transporter gene *Slc2a4* (Glut-4) was less strongly expressed in the iBRB than in the BBB, whereas *Slc16a1*, another member of the Slc family, was enriched in the iBRB [86].

**Lung:** The continuous vascular endothelium of the lung comprises different types of specialized ECs that can be distinguished from the ECs of other organs. At the interplay with the extremely thin (<0.1 µm) type I alveolar epithelial cells, the capillary ECs of the lung are responsible for gas exchange. Together with these alveolar epithelial cells and the basement membrane between both cell layers, capillary ECs form the blood–air barrier. Recently, it has been found that the capillary ECs of the lung can be classified into different groups, the “aerocytes”, large cells with “swiss cheese-like” pores, specialized for gas exchange, and the “general capillary cells (gCaps)”, functioning as stem/progenitor cells which are specialized to regulate vasomotor tone [13]. Both cell types seem to be able to regulate each other, as aerocytes express ligands (Apln and Kitl), that can be detected by cognate receptors on gCaps (Aplnr and Kit), and, conversely, gCap cells express ligands (Edn1 and Vegfa), that can be sensed by cognate receptors on aerocytes (Ednrb and Kdr) [13]. An additional EC type, so-called “Car4-high” pulmonary ECs, has been described shortly before, which seems to detect signals of alveolar type I cells in response to acute lung injury [89]. Regarding metabolic gene signatures, pulmonary ECs show increased expression of genes associated with the metabolism of cyclic adenosine triphosphate (cAMP), in comparison to ECs from other organs [11], possibly associated with the regulation of the pulmonary endothelial barrier [90]. Pulmonary arterial and arteriolar ECs also feature a distinct phenotype related to archaic/conserved stress response pathways. While the activation of the tumor suppressor p53 initiates a vasculoregenerative program in pulmonary arterial and arteriolar ECs [91,92], in other vascular systems, endothelial p53 was detrimental to vascular regeneration and homeostasis [93,94,95]. The exact underlying mechanisms for a distinct response of a single transcription factor such as p53, e.g., organ-specific interaction partners, dose-response effects or chromatin accessibility, remain the focus of ongoing investigations [91,96].

**Heart:** Cardiac development, but also pathogenic cardiac remodeling, is based on the cross-talk between cardiomyocytes and cardiac ECs [97,98] and it is not surprising that cardiac ECs can be distinguished from the ECs of other organs by distinct expression signatures as revealed by RNAseq [99]. Lother and colleagues found the *Meox2/Tcf15, Fabp4* and *Cd36* signaling cascade to be highly upregulated in cardiac ECs, compared to non-selected heart tissue or the ECs of the brain, kidney, lung and skeletal muscle [99]. These genes are part of a signaling cascade that regulates fatty acid uptake. This is particularly important, as myocardial contraction consumes huge amounts of ATP-mediated energy provided by mitochondrial fatty acid oxidation [100].

**Adipose tissue**: Adipose tissue, both brown and white fat, is a well-vascularized energy depot. The energy is stored in lipid droplets within adipocytes as the major cell type of this tissue. PPARγ is an important regulator of adipose differentiation and crucial for the ability to store lipids. The vascular ECs of adipose tissue have been proposed to possess the capability to de-differentiate and give rise to adipocyte progenitor cells via endothelial-pericytic intermediates [101,102]. This idea has recently been challenged by a study showing that exogenous fatty acids stimulate PPARγ activation in the microvascular ECs of adipose tissue, which indeed leads to increased fatty acid transport and accumulation of small lipid droplets. However, the transdifferentiation of ECs into adipocyte progenitors has not been observed. Instead, the highly specialized microvascular ECs seem to play a very important role in adipose tissue lipid uptake by secreting PPARγ ligands [103].

**Intestines:** Being colonized by a myriad of microorganisms, the intestines are a potential gateway for pathogens into the bloodstream. Therefore, in close interaction with enteric glial cells and pericytes, the endothelial cells of the intestines form a so-called gut-vascular barrier (GVB), preventing translocation of microbiota [104]. Intestinal ECs possess tight junctions composed of occluding, zonula occludens-1, cingulin and junctional adhesion molecule-A, as well as adherens junctions formed by vascular endothelial cadherin and ß-cadenin [104]. Apart from their gate keeper function, intestinal ECs need to be able to absorb nutrients. Consequently, genes involved in the uptake and metabolism of fatty acids (*Aqp7, Tcf15, Cde6, Fabp5*) have been found to be upregulated in a subpopulation of small intestine ECs [11].

#### 2.2.2. Fenestrated Endothelium

Fenestrated capillary ECs possess transcellular pores lined by plasma membrane (called fenestrae) and a continuous basal lamina with small gaps. Water, solutes and some peptide hormones can diffuse through the fenestrae without the need of being actively transported through the cell. Fenestrated ECs can be found in the choroid plexus of the brain, in endocrine organs and within the glomeruli and tubuli of the kidney [13]. Endothelial fenestrae often contain membrane spanning plasmalemmal vesicle associated protein (VP1), forming spore-like structures spanning the fenestrae close to their base. These structures, called diaphragms, can be found in most but not in all fenestrated ECs (see below).

**Kidney**: A renal EC-specific transcription pattern has been observed upon culturing primary kidney ECs [105]. Many of the upregulated genes upon cell culture are directly linked to the development of complex renal structures [105]. They include transcription factors, such as EBF1 and PAX2, critical for the development of glomeruli and branching morphogenesis, respectively [106,107,108], as well as a large set of kidney-specific homeobox transcription factors [107]. Overall, renal ECs strongly differ from the ECs of other organs such as the brain, heart, lung and skeletal muscle [109]. Anyhow, not all ECs within the kidney are equal. In fact, the kidney represents one of the organs with the highest intra-organ EC heterogeneity due to a multitude of functions and the highly complex vascular structure [110]. The microvascular endothelium within the glomeruli of the renal cortex is responsible for the ultrafiltration of blood plasma, whereas solute exchange and water resorption take place at the peritubular capillaries in the medulla [111]. Consequently, the ECs of the renal glomeruli, cortex and medulla harbor distinct EC populations (gRECs, cRECs and mRECs) [69]. The functional diversity of renal ECs is reflected by a highly distinct morphology of the different EC subpopulations, as revealed by electron microscopy. Capillary gREC fenestration is induced by tightly regulated expression of podocyte-derived VEGF-A [112]. Mature gREC fenestrae have been shown to lack diaphragms [113], whereas peritubular cRECs contain diaphragms [114]. Consequently, the PV1-encoding gene *Plvap*, a typical marker of fenestrated ECs, is not expressed by glomerular capillaries but by cortical peritubular capillary ECs [69]. Single cell RNAseq showed distinct gene expressions signatures with upregulation of *Ehd3, Cyp4b1* and *Tspan7* in gRECs [110,115,116,117], *Igf1* and *Cd36* in mRECs [110,115] as well as *Igfbp3* and *Npr3* in cRECs [110]. The three main populations of renal ECs can be further subclustered, according to their exact origin (arteriolar, venous, capillary, etc.), into five gREC subclusters, nine cREC subclusters (one of which interestingly showing an interferon-response phenotype) and ten mREC subclusters [109]. In order to drive filtration, the hydraulic pressure and flow rates in glomerular capillaries are substantially higher than in systemic capillaries and the filtration of protein-free water/small solutes concentrates large plasma proteins, raising the viscosity. Therefore, shear stress, which is positively related to flow and viscosity, is higher in glomerular capillaries than in systemic capillaries [69] leading to upregulation of the shear-stress-induced gene *Pi16* in capillary gRECs [110]. To maintain perfusion pressure, gRECs further express genes such as *Gata5*, which is involved in NO signaling pathways [118] and *Tbx3*, which regulates renin secretion [119]. The different EC populations within the kidney are indeed affected differently by some pathologies. Hypertension, for example, has been reported to induce, primarily, the rarefaction of peritubular capillaries [120]. Dysfunction of glomerular ECs, on the other hand, is involved in the development of glomerular sclerotic diseases, such as focal segmental glomerulosclerosis and diabetic nephropathy [121]. In atypical haemolytic uraemic syndrome, gene mutations lead to reduced factor H binding to glomerular endothelial heparan sulfate [122].

**Choroid plexus**: Unlike the BBB-associated ECs, the ECs of the choroid plexus are fenestrated and not connected by tight junctions. Fenestrae of choroid plexus ECs contain diaphragms [123]. The blood-cerebrospinal fluid barrier is mainly composed of the associated epithelial cells and their tight junctions. Located in the cerebral ventricles, the choroid plexus is responsible for secreting the cerebrospinal fluid. The choroid plexus is also home to many immune cells. Multiple studies have compared the gene expression signatures of healthy ECs or dysfunctional ECs within the choroid plexus [124,125,126]. As for the BBB, many important transporters, such as various ABC transporters and solute carriers, were shown to be strongly expressed [124,125,126]. A transcriptome analysis of choroid plexus endothelial cells revealed upregulated expression of *Plvap*, a typical marker of fenestrated endothelium [127] with high abundance of EC caveolae [11]. Due to their functional and morphological differences compared to other ECs and their unique expression profile (a combination of genes being typically expressed in fenestrated ECs and genes being upregulated in the ECs of the BBB), choroid plexus ECs are clearly distinguishable from most other EC populations of the body.

**Endocrine glands:** A fenestrated endothelium can be found in some endocrine organs including thyroid and pituitary gland. The capillaries of the thyroid gland are heavily fenestrated [128] and wrapped by PDGFRβ-expressing pericytes [129]. Thyroid follicular cells and microvascular ECs form angiofollicular units that control endocrine function [128]. The adenohypophysis of the pituitary gland is the most strongly vascularized tissue in mammals [128,130]. Here, fenestrated capillaries form a plexus at the top of the pituitary stalk. Fenestrae of thyroid gland and pituitary gland ECs contain VP1 diaphragms [129]. Gene expression data enabling further distinction of endocrine gland ECs from other fenestrated ECs are still lacking.

#### 2.2.3. Discontinuous Endothelium

Discontinuous endothelium is the least dense endothelial barrier; therefore, it provides the highest permeability for large molecules and circulating cells through gaps in the endothelial layer and the basement membrane. A discontinuous endothelium can be found in sinusoids from liver, bone marrow and spleen [5].

**Liver:** The vascular system of the liver consists of a network of arterioles, sinusoids and venules. Liver sinusoidal ECs (LSECs) are specialized cells interconnecting the blood stream, hepatocytes and stellate cells [131]. On their luminal sinusoidal side, these cells are exposed to a mixture of oxygen-rich arterial blood and the blood from the portal vein originating from the intestines which contains nutrients, metabolites and hormones, such as insulin and glucagon. On the abluminal side, LSECs interact with hepatocytes that are crucial for protein, lipid and glucose metabolism [132] as well as with hepatic stellate cells. Fenestrated LSECs, with their discontinuous basement membrane, represent the most permeable EC barrier for macromolecules within the mammalian body [133]. LSEC differentiation, which is driven by hepatoblasts, is accompanied by a gradual loss of typical cell markers of continuous ECs, including CD31 and CD34, while markers of adult sinusoidal cells, including CD4, CD32 and the intracellular adhesion molecule-1 (*ICAM-1*), are acquired during differentiation [131]. LSECs have minimal basement membranes and loosely organized cell junctions. LSEC pores range from 50 to 150 nm in diameter [134,135] depending on their localization within the liver. LSEC pore number and size change rapidly in response to dietary intake or intoxication [136]. Larger but fewer pores are found in the periportal region, whereas pores in the centrilobular region are more frequent but smaller [135,137]. The main physiological and immunological functions of LSECs include filtration, endocytosis, antigen presentation and recruitment of leukocytes [136]. The expression of several scavenger receptors, a diverse family of pattern recognition receptors, is upregulated in LSECs. Belonging to the group of toll-like receptors, scavenger receptors are highly conserved during evolution and play an important role in the innate immune response [138]. Further, LSECs regulate adaptive immune responses through antigen presentation to T cells [134]. Overall, LSECs strongly express vessel endothelial hyaluronic acid receptor (LYVE1), prosperohomobox protein 1 (PROX1) and ICAM3-grabbing non-integrin (LSIGN) [136]. As in other organs, the microvascular ECs of the liver can be further divided into different subgroups according to their gene expression signatures. LSECs in the liver acinus zone1 express high levels of CD36 and lower levels of LYVE1, whereas LSECs of zone 2 and 3 express low levels of CD36 and high levels of LYVE1, as well as of CD31 [139].

**Bone Marrow:** Bone marrow is a highly proliferative tissue producing an estimated 500 billion cells per day [140]. The hematopoietic stem cell niche is heavily vascularized and the vascular endothelium represents an essential part of the bone marrow microenvironment, contributing to homeostasis and hematopoietic regeneration [141]. It can be assumed that hematopoietic stem cells, as well as vascular ECs, directly influence the gene expression pattern of their respective counterpart [142]. As in other organ systems, arterioles, sinusoids and venules of the bone marrow differ in function and phenotype. Bone marrow ECs are the major source of the Notch ligand DII4, which plays an essential role in hematopoietic stem cell differentiation [143]. Interestingly, bone marrow arterial ECs (BMAECs) rather than bone marrow sinusoidal ECs (BMSECs) secrete the vast majority of detectable EC-derived stem cell factors necessary to maintain the hematopoietic stem cell niche [144]. BMAECs can be distinguished from BMSECs by differential expression of podoplanin (*Pdpn*) and *Sca-1* and both subtypes seem to be derived from independent pre-specified EC precursors [144].

#### 2.2.4. High Endothelial Venules

Postcapillary high endothelial venules (HEVs) comprise a specialized form of ECs with a cuboidal morphology and thickened basement membrane which can be found in lymph nodes and tertiary lymphatic structures [145,146]. Although being directly associated with lymphatic structures, HEV ECs belong to the vascular system [147,148,149]. HEVs function as entry port for lymphocytes from the blood into the lymphatic tissue [14] and HEV ECs express a set of molecules that enables lymphocyte interaction and adhesion such as CCL21 [150], ICAM-1 [151] as well as the HEV-typical peripheral node addressin (PNAd) [145] and mucosal vascular addressin cell adhesion molecule (MAdCAM) [151]. In order to control lymphocyte trafficking, HEV ECs form pockets that serve as lymphocyte “waiting areas” in which lymphocytes reside for several minutes before entering the lymph node parenchyma [152]. Consequently, HEVs rapidly expand upon immune stimulation and inflammation [153]. HEVs have also been reported to be present in different solid tumors [154] and tumor-associated HEVs have been proposed to play an important role in lymphocyte infiltration into tumor tissue [153]. Further, development of HEV-like blood vessels and their association with perivascular lymphocytes has been observed in non-lymphoid tissue in multiple chronic inflammatory diseases as reviewed in [153].

## 3. Endothelial Heterogeneity in Response to Stress

**Figure 2:**
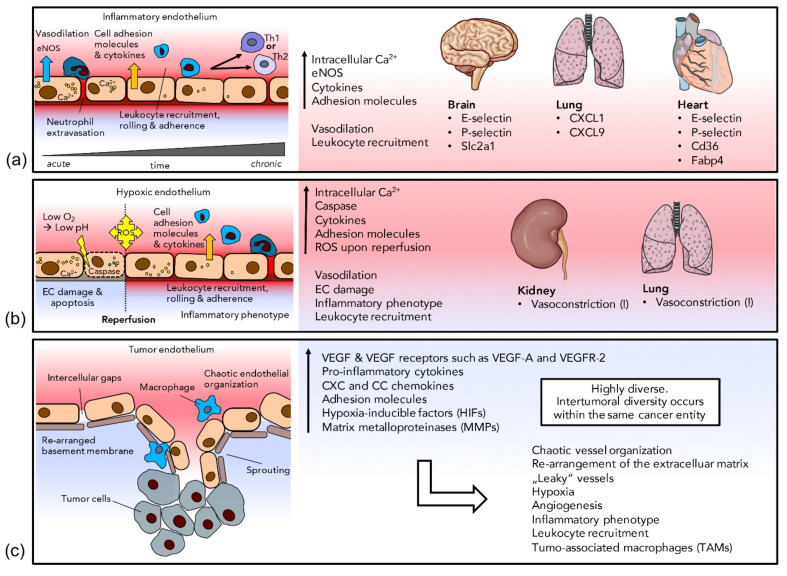
EC heterogeneity in response to stress. (**a**) During the acute inflammatory response, increased intracellular calcium activates eNOS and leads to vasodilation. Attracted neutrophils transmigrate through the endothelial layer. Upregulation of adhesion molecules and pro-inflammatory cytokines leads to further leukocyte recruitment. The attraction of either Th1 or Th2 cells determines the further inflammatory process. During inflammation, some organs, such as brain, lung and heart preferentially express certain adhesion molecules, cytokines or transporters. (**b**) The response to hypoxia shows some similarities to the inflammatory response. Intracellular calcium increases and, in most organs, leads to vasodilation. The small vessels of the kidney and the lung conversely react with vasoconstriction. Low oxygen and low pH lead to excessive amounts of calcium, resulting in cell damage and caspase-mediated apoptosis. Further damage is induced by reactive oxygen species (ROS) upon reperfusion. Increased expression of cell adhesion molecules and pro-inflammatory cytokines leads to leukocyte recruitment and fosters an inflammatory phenotype. (**c**) The tumor endothelium is highly angiogenic and appears chaotic. The extracellular matrix is rearranged and the endothelial layer is leaky, allowing the transmigration of tumor-associated macrophages and other leukocytes to take place. Tumor ECs express VEGFR-2, pro-inflammatory cytokines, cell adhesion molecules, hypoxia-induced factors (HIFs) and matrix metalloproteinases. Tumor ECs are highly diverse, even within the same cancer entity. This figure contains artwork components of Servier Medical Art.

As illustrated above, the endothelium is a highly versatile organ able to swiftly react to external stimuli by changing morphology and function in order to adapt to changing physiological conditions. Under pathological conditions, the endothelium acquires a dysfunctional disease-promoting phenotype. Activated ECs during inflammatory events can be clearly distinguished from the resting endothelium and dysfunctional ECs differ from healthy ones. In this section we discuss three major pathological changes within the vascular endothelium. A schematic overview of endothelial heterogeneity in response to stress can be found in Figure 2.

### 3.1. Inflammation

ECs are extremely important players in inflammatory processes and their first response to acute inflammatory triggers is rapid [155]. ECs mediate increased blood flow to inflamed tissues, recruit circulating leukocytes and enable their extravasation. Activation of G protein-coupled receptors in response to acute inflammation leads to an increase in cytosolic calcium, which elevates the arachidonic acid concentration via the activation of phospholipase A2. In turn, arachidonic acid is converted to PGI2 by cyclooxygenase 1 (COX1) and prostacyclin synthase [155]. PGI2 acts as a strong vasodilator [156]. Vasodilation is augmented by increased NO levels due to calcium-activated endothelial nitric-oxide synthase (eNOS). Leukocyte recruitment is also facilitated by calcium increase via the phosphorylation of myosin light chain (MLC). Activated MLC leads to P-selectin translocation from Weibel–Palade bodies to the cellular luminal surface, attracting circulating neutrophils [156]. The interaction of P-selectin with platelet-activated factor (PAF) initiates neutrophil extravasation [157]. In addition to this first rapid response mediated by G protein-coupled receptors, a second type of activation takes place at the gene expression level. During acute inflammation, ECs are stimulated by tumor necrosis factor (TNF-α) and interleukin-1 (IL-1), eventually leading to the activation of pro-inflammatory transcription factors, such as nuclear factor-kB (NF-kB), via various signal cascades [158]. The NF-kB-mediated inflammatory EC signature includes increased expression of leukocyte adhesion molecules, such as E-selectin [159], intercellular adhesion molecule 1 (ICAM1) and vascular cell-adhesion molecule 1 (VCAM1) [160] as well as several chemokines, such as CXCL8 and CCL2 [160]. In pulmonary arterial ECs, the transcription of cytokines interleukin(IL)-6 and IL-8 is a fast process and occurs within minutes [92].

Upon continuous inflammatory stimulation, ECs change towards a more chronic inflammatory phenotype. Either CXCL10 becomes upregulated sustaining an increased E-selectin level, which, in turn, recruits TH1 cells [161], or CCL26 and VCAM1 become upregulated, leading to the preferential recruitment of TH2 cells [155]. ECs also express MHC class I and II molecules; therefore, they have been proposed to be involved in antigen presentation and activation of CD4+ and CD8+ memory T cells [162]. Recently, organ-specific differences have been observed in EC response to inflammation [163]. While the classical leukocyte adhesion molecules P-selectin and E-selectin were induced in brain and heart ECs, lung ECs were shown to increase the expression of chemokines, such as CXCL1 and CXCL9 [163]. Further, brain ECs were shown to increase expression of the glucose transporter Slc2a1, attesting to increased glucose consumption upon inflammation [164], while cardiac ECs upregulate expression of fatty acid metabolism genes Cd36 and Fabp4, attesting to an increased metabolic rate of the myocardium [165].

### 3.2. Ischemia/Hypoxia

ECs are particularly sensitive to ischemic conditions and subsequent reperfusion and react with hypoxia-induced changes in gene expression, which results in an inflammatory phenotype (see above) and EC dysfunction [166], including the overexpression of E-selectin, ICAM-1 and receptor for advanced glycation endproducts (RAGE) as well as activation of NF-kB. Low pH and lactate accumulation during ischemia further leads to massive amounts of cytosolic Ca^2+^ in EC due to calcium leakage from the endoplasmatic reticulum (ER) [167]. Excessive Ca^2+^ levels, in turn, dysregulate vascular tone [168], severely damage ECs and promote apoptosis by cleavage of ER-bound caspase 12 [169], attesting to the complexity and spatio-temporal context dependency of calcium signaling [170]. In contrast to the vasodilatory effect of calcium upon inflammation, in some organs, such as the lung and the kidney, ischemic or hypoxic ECs even mediate vasoconstriction [171,172]. The exact pathways of hypoxia-mediated vasoconstriction have not been fully elucidated yet [172], but adenosine, as well as altered expression of kallikrein, ACE and neutral endopeptidase, might play a role in the renal vasculature [173,174]. Further, there is reduced NO in the renal vasculature upon hypoxia, which driven by a loss of endothelial nitric oxide synthase function, probably resulting from oxidization of tetrahydrobiopterine (BH4), a co-factor of eNOS [171]. Since NO is not only a potent vasomodulator but also an inhibitor of neutrophil activation and adhesion, reduced NO may foster the inflammatory phenotype observed upon ischemia [175]. Reperfusion of ischemic tissue increases levels of reactive oxygen species and causes oxidative stress which, again, promotes an inflammatory phenotype including overexpression of E-selectin, P-selectin, ICAMs [176] and activation of NF-kB [177].

### 3.3. Cancer

Rapid proliferation of tumor cells, tumor formation and tumor growth depend on a sufficient oxygen and energy supply. To meet their demands, tumor cells highjack the vascular system, induce angiogenesis and facilitate the growth of their own vascular network [178]. Early tumor development results in a hypoxic milieu, which leads to the expression of hypoxia-inducible factor (HIF) and proangiogenic chemokines, as well as vascular endothelial and platelet-derived growth factors (VEGF and PDGF) [178]. During angiogenesis, navigating tip cells and proliferating stalk cells are induced upon binding of VEGF-A to the endothelial VEGF receptor 2, which leads to the formation of novel vascular sprouts [178]. In healthy tissue, the expression of pro- and anti-angiogenic factors is tightly regulated [179,180]. However, in tumor tissue, the expression of VEGF and a number of anti-angiogenic factors is uncoordinated and out of balance [28]. Tumor blood vessels show an abnormal morphology, lacking the classical vessel hierarchy [181]. The endothelial network appears chaotic and the EC layer is defective with a discontinuous basement membrane. In tumor tissue, vascular endothelium—normally consisting of a single layer of ECs—can be turned into EC multilayers. Further, excessive branching of tumor vessels happens randomly, which, together with pathological intercellular gaps in the endothelium, leads to tumor areas with undersupply of blood [182] which, again, promotes hypoxia. Hypoxia in tumors is associated with the development of a metastatic disease state [183]. Tumor ECs not only regulate blood supply, but are also a key to the tumor’s immune escape [182]. ECs represent the first line of contact for circulating immune cells and guide adhesion, rolling and extravasation of immune cells, eventually leading to infiltration into the adjacent tissue [184]. However tumor ECs, promote an immunoprivileged environment by repressing the response to inflammatory activation and the expression of leukocyte adhesion molecules [185]. Nagy et al. classified the vascular network of the tumor into six vessel types according to their morphology: (1) mother vessels, which are enlarged, tortuous, thin-walled, pericyte-poor hyperpermeable sinusoids; (2) normal capillaries; (3) glomeruloid microvascular proliferations, which are tangles of tiny vessels immersed in a complex mixture of irregularly ordered pericytes and extensive multilayered basement membrane; (4) vascular malformations being large vessels with an irregular coat of smooth muscle cells; (5) feeder arteries;(6) draining veins, which are greatly enlarged, tortuous smooth muscle-cell-coated vessels that supply and drain the complex of angiogenic blood vessels [186]. Tumor EC diversity has recently been resolved in even further detail and seems to be site-specific [187]. Applying single cell RNAseq, Goveia et al. identified as many as 10 different tumor vascular EC subtypes in non-small cell lung cancer (NSCLC) patients [187]: Arterial EC expressing genes, involved in vascular integrity, hemostasis and vasotonus; postcapillary venous EC-expressing genes, involved in leukocyte recruitment, tissue perfusion and blood pressure; type I and type II alveolar capillary ECs, differentially expressing VWF and EMCN (endomucin); scavenging capillaries with upregulated expression of scavenger receptors; activated and intermediate capillaries, expressing EC activation markers; tip cells, expressing genes involved in EC migration, matrix remodeling and VEGF-A signaling; immature tumor ECs, which are similar to tip cell but additionally express genes involved in the maturation of newly formed vessels, barrier integrity and Notch-signaling; activated postcapillary vein EC with upregulated immunomodulatory factors and ribosomal proteins [187]. Comparing the NSCLC samples with samples from other tumor entities or species, tip tumor EC markers, such as ANGPT2, APLN, FSCN1, PGF, PLXND1, ADM, PDGFB and CXCR4, seemed to be the only conserved pattern across species and models [187]. High-level expression of the angiogenic tip EC signature also seems to be negatively correlated with overall survival of squamous (but not adenocarcinoma) NSCLC patients [187]. Another very important feature of tumor ECs is related to formation and rearrangement of the extracellular matrix (ECM). Single cell sequencing of breast-cancer-associated ECs revealed upregulation of ECM-associated genes [188], which is in line with previous studies reporting upregulated expression of ECM-associated genes [189] such as MMP9 [190], TIMP1 [190], and MMP14 [191] in ECs of different tumor entities. In line with increased angiogenesis within the vascular network of the tumor, it is not surprising that there is continuous need for ECM rearrangements.

## 4. Selective Targeting of Distinct EC Subpopulations

Selective targeting and manipulation of distinct EC subpopulations helps to further assess their role and function under physiological conditions and may be therapeutically beneficial in pathological settings. While cytotoxic or antiangiogenic therapies might help to treat cancer, the opposite—namely, the induction of angiogenesis to allow reperfusion to happen—might be indicated under other circumstances, such as in ischemic tissue. Another example is the tumor-suppressor p53, which mediates apoptosis in some cells (i.e., tumor cells), while it acts as pro-survival factor in others (i.e., pulmonary EC). Therefore, most interventions require highly selective targeting.

**Figure 3:**
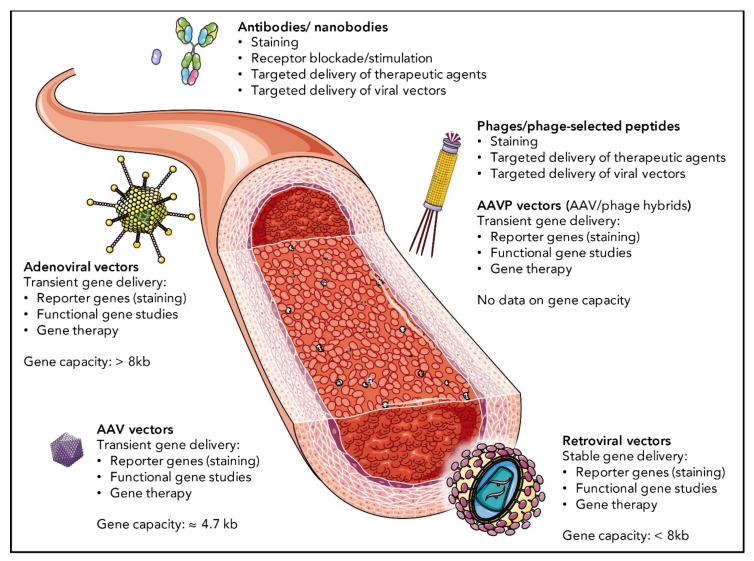
Different approaches of selective EC targeting. Antibodies, nanobodies and phage-selected peptides, as well as different kinds of viral vectors, can be used to specifically target distinct EC populations. Possible applications and limitations of the different targeting approaches are indicated. This figure contains artwork components of Servier Medical Art.

Although truly selective targeting has been achieved in some instances, many of the attempts described below more broadly aim at the angiogenic endothelium, assuming a preferential targeting of tumor ECs or other diseases associated with hyperproliferative endothelium. As angiogenic markers are expressed by ECs in the intended situations as well as in others (examples are given in Section 2.2), many VEGF(R)-targeted approaches may bear the risk of side effects in therapeutic settings. In this section, we discuss promising EC targeting strategies and provide examples of successful EC targeting in vitro and/or in vivo. A schematic overview of the different targeting approaches and possible applications is shown in Figure 3. A comprehensive list of all examples discussed in this review is given in Table 1.

### 4.1. EC Targeting by Antibodies and Nanobodies

In principle, available antibodies against common endothelial surface markers, such as PECAM-1 (CD31), vWF, or VE-cadherin (CD144), are not restricted to diagnostic or research purposes and may also be applied for medical interventions, when coupled to therapeutic agents. The same is true for targeted nanobodies (small fragments of camelid heavy chain antibodies) [244]. However, very few antibodies against EC surface molecules enable to distinguish between different EC subpopulations or cellular (disease) statuses. The monoclonal antibody clone MECA-79 that recognizes carbohydrates on PNAd, a molecule specifically expressed by HEV EC, is an example of such highly selective EC targeting. Applying MECA-79 antibody in a sheep model of asthma inhibited leukocyte accumulation in bronchoalveolar lavage fluid and ameliorated late-phase airway response and airway hyperresponsiveness induced by airway allergen challenge [204]. Further, nanoparticles coated with MECA-79 antibodies have been used to transport anti-CD3 to lymph node HEV, which prolonged cardiac allograft survival in a mouse model [203]. Antibodies against angiotensin converting enzyme (ACE) represent another promising targeting approach, although ACE expression is, by far, not restricted to pulmonary ECs [76]. Instead, ACE is ubiquitously expressed in all EC subtypes and even more abundantly expressed in spermatids, enterocytes and intestinal endocrine cells. However, upon intravenous injection, ACE antibodies seem to enrich in the lung; thus, they have successfully been used to target pulmonary ECs. Catalase coupled to ACE-antibodies, for example, has successfully been used to condition the pulmonary endothelium before lung transplantation in rats [218,219] and ACE antibodies have also been used to target therapeutic viral vectors to the pulmonary endothelium, as discussed further below. Another example for antibody-guided EC targeting is VCAM-1, which is upregulated upon cytokine induction; therefore, it enables the preferential targeting of inflammatory events in the vasculature. Specific nanobodies targeting VCAM-1 have been used for imaging atherosclerotic lesions by single-photon emission computed tomography or positron emission tomography in preclinical models [205,207]. Coupled to microbubbles, VCAM-1 nanobodies have also been used for contrast-enhanced ultrasound molecular imaging [208]. Antibodies against plasmalemmal vesicle-associated protein (PLVAP) [245], a molecule specifically localized in endothelial caveolae which is upregulated in multiple diseases including brain tumors [246,247], traumatic spinal cord injury [248], and acute ischemic brain disease [249], have been used in different preclinical studies for therapeutic purpose. SOD-conjugated anti-PLVAP antibody, for example, alleviated endotoxin-mediated inflammation in mice [206], and conjugates with PGE2 were shown to be beneficial in mice with pulmonary fibrosis [220]. Several studies report on the therapeutic targeting of other more broadly expressed EC surface proteins by antibodies or nanobodies. Vascular endothelial growth factors (VEGF) and their broadly expressed cognate receptors (VEGFR) play an important role in angiogenesis and their expression is upregulated in highly angiogenic sites, such as the tumor microenvironment or in the retina in the event of age-related macular degeneration (AMD), diabetic retinopathy and retinal vein occlusions [250].Various groups generated nanobodies against vascular endothelial growth factor receptor-2 (VEGFR-2), which might serve as anti-angiogenic agents [251,252]. Coupled to a light-activatable photosensitizer, such VEGFR-2-targeted nanobodies have recently been shown to enhance photodynamic therapy in VEGFR-2-expressing cells in vitro [253]. However, much like other clinically approved anti-angiogenic antibodies directed against VEGF, such as bevacizumab [254], aflibercept [255] or ranibizumab [256], VEGFR-2 targeted drugs are not selective for the vasculature, as VEGFR-2 expression is not limited to ECs. In fact, pancreatic duct cells, retinal progenitor cells, megakaryocytes and different cancer cells are known to express VEGFR-2 as well [257]. Further, VEGFR are expressed by ECs outside of the angiogenic setting. In the kidney, for example, VEGFR-2 and its co-receptor neuropillin-1 are expressed by glomerular and peritubular capillaries [258]. Soluble VEGFR-1, on the other hand, has been proposed to play a role in renal disease associated with preeclampsia [259]. Consequently, VEGF signaling has been linked to glomerulopathies [260] which indicates a risk for severe side effects of VEGF(R)-targeted anti-angiogenic therapies. To further increase specificity, the above-mentioned clinically approved and many more VEGF(R)-targeted anti-angiogenic antibodies have been applied locally to treat, e.g., AMD or other retinal disorders [261]. Antibodies against the most commonly expressed EC surface markers, such as PECAM-1 (CD31), can be used for therapeutic approaches, as long as selective targeting is not required. Coupled to catalase, PECAM-1 antibodies have been used to reduce oxidative stress during lung transplantation in rats [226] and pigs [227] and as a treatment for experimental acute lung injury in a mouse model [221,222]. Experimental acute lung injury has also been successfully treated by PECAM-1 antibody fragments fused to thrombomodulin [223]. In another approach, PECAM-1 antibodies have been fused to urokinase-type plasminogen activator (uPA) to treat experimental conditions in mice, including pulmonary embolism [224,225] and ischemic stroke [199]. However, this review focuses on selective targeting of EC subpopulations and examples of highly specific antibodies are rare. New antibodies can be screened by variants of the phage display technique, in which single chain antibody fragments or full-length IgG antibodies are displayed on bacteriophages. After binding immobilized target structures, successful phage candidates can simply be amplified in *E. coli* bacteria and further analyzed for specificity [262]. Another very successful method to identify vascular targeting molecules is the in vivo screening of phage-displayed peptide libraries.

### 4.2. EC Targeting by Phage-Displayed Peptides

Before next generation sequencing and microarrays became broadly available to tackle the vast EC diversity, in vivo screening of highly diverse peptide libraries displayed on bacteriophages revealed surprisingly specific homing of certain peptides or peptide motifs to individual regions of the vasculature upon intravenous library administration, leading to the concept of “vascular ZIP codes” [201]. Phage display has emerged as one of the most powerful techniques to identify vascular targets and organ-specific peptide homing could be observed within the vasculature of different species, including mice, rats and humans, in many instances, even enabling the distinction between healthy and diseased stage (e.g., tumor ECs vs. non-tumor ECs). Phage-selected peptides can be coupled to or incorporated into an immense variety of molecules, including molecular dyes, therapeutic agents or viral vectors. The selective homing of phages, in several cases, outperforms the moderate selectivity of antibodies described in the previous paragraph. One intriguing early example is the phage-selected double-cyclic peptide CDCRGDCFC (RGD-4C) homing to the tumor vasculature of mice upon binding to αv-integrins via its “RGD” motif [232,233]. αv-integrins are preferentially (although not exclusively) expressed on the ECs of the tumor vasculature and on tumor cells. Coupled to the anti-cancer drug doxorubicin, tumor EC-targeted peptides harboring the “RGD” motif have successfully been used to treat tumor xenografts in a mouse model [263]. The RGD-4C motif has also been used to target hybrid vectors of fd-tet bacteriophage and recombinant AAV, so called AAVPs, to the tumor vasculature [237,238], allowing for gene expression in the target cells in vivo. The AAVP platform has recently been improved to avoid degradation during pre- and postinternalization [264]. Further phage library screenings yielded the “internalizing-RGD” (iRGD) peptide, which, in addition to the integrin-binding RGD motif that mediates vascular attachment, releases a cryptic “CendR motif” (RGDK/R) upon cleavage, allowing for cell internalization via Neuropilin-1 [234]. The iRGD peptide has been tested for its ability to improve cancer therapy in multiple preclinical settings. Successful examples include improved lymphocyte infiltration in a xenograft mouse model of gastric cancer [235], improved efficacy of the anti-cancer membrane-active peptide HPRP-A1 [236], or improvement of tamoxifen-induced killing of estrogen receptor positive breast cancer cells [265]. Due to its highly promising therapeutic potential, the iRGD peptide has recently been tested in a first clinical phase I trial in combination with Nab-paclitaxel and Gemcitabine and the results are expected to be published soon (ClinicalTrials.gov, Identifier: NCT03517176). Similar affinity to tumor EC has been shown for the “NGR” motif identified by the same group [263], which targets aminopeptidase N, also known as CD13 [266], a molecule upregulated within tumor EC of mice and humans. Coupled to cytokines TNF and IFNγ, the “NGR”-harboring peptide could be used to elicit anti-tumor effects in mice [267,268]. Other peptides homing to the tumor neo-vasculature of mice or human lung cancer biospecimen, as well as vascular growth factor-stimulated HUVECs, include SVSVGMKPSPRP (SP5-52), and SVSVGMKGGGRP (MP5-52) [269]. Coupled to doxorubicin, these peptides increased therapeutic efficacy in a xenograft mouse model, as has been described for the RGD-harboring peptides. In addition, the heparin-binding domain of VEGF (CSCKNTDSRCKARQLELNERTCRC) is able to confer targeting to tumor ECs when displayed on bacteriophages [270].

Impressive examples for the targeting of organ-specific ECs are the “GFE-1” peptide (CGFECVRQCPERC) [211], specifically homing to the murine pulmonary vasculature by binding to membrane dipeptidase [212], or the peptides VNTANST and QPEHSST, homing to the vasculature of lung and brain, respectively, when applied to Wistar Kyoto rats [198]. Later, another phage peptide homing to murine cerebral vasculature, CAGALCY, has been discovered [193]. Specific targeting of brain ECs by phage-displayed peptides was also achieved in a mouse model of the lysosomal storage disorder MPS VII. Here, the authors were even able to identify peptides that distinguish between the cerebral vasculature of healthy and diseased mice [197]. More recently, phage-displayed peptides harboring the “FRW” motif were shown to specifically bind to the junctions of brain endothelial cells but not to the junctions of retina endothelial cells, although these cells have always been considered closely related [200]. The kidney and the heart represent additional organs that have been successfully targeted via the vasculature. The peptides CLPVASC [229] and PKNGSDP [230], as well as DSHKDLK [230] were shown to specifically target the kidney’s vessels in mice and rats, whereas the peptides CRPPR, CARPAR, CKRAVR, CRSTRANPC and CPKTRRVPC, confer specific homing to cardiac ECs in mice [192]. In addition, phage display has enabled identification of vascular-targeted peptides for white fat presenting the CKGGRAKDC motif homing to the membrane protein prohibitin [243]. Conjugating the CKGGRAKDC peptide to the apoptosis-inducing peptide (KLAKLAK)2 was sufficient to ablate white fat in obese mice [243]. Less specific for EC subpopulations and more broadly relevant to vascular trafficking in general was the discovery of phage peptides displaying the “LLG” motif. “LLG” peptides were shown to block leukocyte adhesion to ICAM-1 and von Willebrand factor by specific binding to β2 integrin in an in vitro model [271]. The enormous potential of phage display is not limited to rodents. A single screening round of a phage library in a deferred human organ donor yielded multiple highly specific EC-targeted peptides, including the peptide CHGGVGSGC, homing to the vessels of the skin, or the CGRRAGGSC peptide, homing to vessels of the prostate [201]. The prostate-homing CGRRAGGSC peptide has later been shown to bind to IL-11Rα by mimicking a receptor binding site within IL-11 [272] and has successfully been used to treat metastatic prostate cancer in human patients [228]. Given the large number of EC-targeted phage peptides discovered during the last two decades, it appears quite likely that additional targeting peptides will become available for large parts of the entire vascular tree.

### 4.3. EC Targeting by Viral Vectors

As an alternative approach, the delivery of transgenes specifically to ECs by means of viral vectors holds great promise for basic research and clinical applications. Viral vectors can be used to deliver reporter genes, such as GFP, for simple detection and identification of distinct EC subpopulations. More importantly, they enable specific overexpression or knockdown of EC genes with very limited off-target effects to study EC gene function in physiological animal models. Eventually, they might be used to treat a broad range of diseases by gene therapy. Adenovirus, adeno-associated virus (AAV) and lentivirus represent the most commonly used vector platforms and there are some impressive cases of EC targeting for each of these viral vector systems upon modifications of the viral surface. As discussed below, in many instances, previously identified targeting molecules such as the above-mentioned antibodies, nanobodies or phage-selected peptides were used to redirect viral tropism towards ECs. Additionally, we discuss promising alternative methods of viral vector-based targeting to the vasculature.

#### 4.3.1. Adenoviral Targeting of ECs

Adenovirus is a common non-enveloped double-stranded DNA virus belonging to the family *Adenoviridae* with a genome size of 25–45 kb, packaged into an icosahedral capsid. Including the protruding fiber proteins, adenovirus measures approx. 90–100 nm in diameter [273]. After attaching to the coxsackievirus/adenovirus receptor (CAR), CD46, heparan sulfate proteoglycan or sialic acids on the cellular surface via its fiber proteins, adenovirus is being taken up by clathrin-coated pit-mediated endocytosis upon binding of the penton base proteins to αv integrin [274]. Following endosomal escape and virion disassembly, the viral DNA enters the nucleus via nuclear pores [275]. Viral gene expression starts without integrating the viral genome into host cell chromosomes. Modern adenoviral vectors are replication-deficient and offer a large packaging capacity for the gene of interest with space for up to 36 kb (3rd generation vectors) [273]. Recombinant adenoviral vectors belong to the earliest and most commonly used vector systems. They have been approved for the treatment of head and neck cancer in China in 2003 (Gendicine) [276] and recently gained attraction as approved vaccinations against Ebola (Zabdeno, Ad26.ZEBOV) [277] and SARS-CoV-2 [276].

Two necessary adenoviral receptors, CAR and αv integrin, play important roles in EC biology and, consequently, are abundantly expressed in ECs. CAR is involved in regulating endothelial mechanotransduction by fluid shear stress [278] and αv integrin plays an important role in growth and survival of newly forming vessels, endothelial cell adhesion, migration, invasion, and proliferation [279]. Therefore, it is not surprising that adenoviral vectors are highly capable of transducing ECs. Indeed, adenoviral vectors have been used in numerous studies to alter and investigate gene functions in primary cultures of human umbilical vein ECs (HUVECs) [280,281,282,283], human or porcine aortic ECs (HAECs/PAECs) [284,285,286,287,288], human saphenous vein ECs (HSVECs), other primary or established EC lines [289,290] as well as organ cultures, such as rabbit carotid arteries [291]. Adenoviral vectors have also been applied in animal models of vascular diseases to test their therapeutic potential or to assess gene function. Examples include overexpression of superoxide dismutase (SOD) in a rat model of hypertension by adenoviral vector infusion into the carotid arteries [292] or overexpression of eNOS in rats with experimental hindlimb ischemia upon intra-arterial vector injection under vascular isolation or under transient vascular occlusion compared to intramuscular injection [293]. In other examples, VEGF delivered to mouse hind limb muscles induced angiogenesis [294] and facilitated choroidal neovascularization when delivered to the subretinal space in rats. Although not directly targeted to ECs, adenoviral delivery of placental growth factor (PIGF) to the adventitia of New Zealand white rabbits led to the induction of angiogenesis [295]. Despite these examples of successful gene delivery to ECs, unmodified adenoviral vectors can neither be considered selective for the vascular endothelium nor do they allow for specific targeting of distinct EC subpopulations. In fact, CAR and αv integrin are expressed quite abundantly on different cell types, many of which show a much higher affinity for adenovirus than ECs do [296]. Off-target accumulation of adenoviral vectors, especially in the liver, strongly limits their applicability for endothelial targeting.

Different methods have been developed to solve this problem by directing adenoviral gene expression more specifically to vascular ECs. One way is the transcriptional targeting by specific promoters or regulatory gene elements. The selective expression of HIF-1α in the vasculature of murine ischemic skeletal muscle, for example, was achieved upon intravenous injection of adenoviral vectors using the preproendothelin-1 promoter (PPE1-3x) [209]. Most approaches of directing adenoviral vectors to ECs involve the modification of viral particles by conjugation or genetic insertion of previously identified targeting molecules. By conjugating bifunctional polyethylene glycol (PEG) to the adenoviral capsid, Ogawara et al. were able to inhibit the interaction between the adenoviral fiber knob and CAR. Introducing E-selectin-specific antibodies or an αv integrin-specific RGD peptide to the second functional group of the PEG molecules enabled the re-direction of adenoviral particles towards activated ECs in vitro and, selectively, to the vasculature in a mouse model of inflammatory skin disease [202,297]. Reynolds et al. were able to target adenoviral vectors to the pulmonary vasculature of rats by coupling bispecific ACE-antibodies to the adenoviral capsid. Transgene expression in the lung was increased 20-fold, whereas transgene expression in the liver was reduced by 80%, compared to unmodified vectors controls [214]. The specificity of transgene expression was further increased by combined transductional and transcriptional targeting. The combination of ACE-targeting by a bispecific antibody fragment and transcriptional targeting by the promoter for vascular endothelial growth factor receptor type 1 (flt-1 promoter) resulted in a 300,000-fold increase in selectivity, comparing the pulmonary vasculature with the liver [217]. An ACE-targeted adenoviral vector delivering the *BMPR2* gene was used by the same group to attenuate pulmonary hypertension in two different rat models (chronic hypoxia and monocrotaline) [215]. Further, applying ACE-targeted bispecific antibodies to an adenoviral eNOS vector, Miller et al. were able to induce hypotensive effects in a rat model of stroke-prone hypertension [214,298]. Phage-selected peptides are another tool to target adenoviral vectors to ECs. Nicklin et al. performed phage display on HUVECs to identify EC-targeting peptides. The most promising peptide, SIGYLYP, was subsequently bound to the adenoviral fiber knob by a bi-specific single-chain Fv antibody. Adenoviral transduction efficacy and selectivity was tested in vitro on HUVECs (target) and human hepatocyte carcinoma HepG2 cells (non-target control) and proven to be superior to the unmodified viral vector [299]. In a second approach, the same group ablated the CAR-binding region of the fiber protein by genetic mutations and genetically inserted the SIGYLYP peptide into the HI loop. Efficacy and selectivity were assessed in vitro as before with significant improvement over the unmodified vector control [300]. Similar approaches include the insertion of phage-selected peptides “NGR” [210], MTPFPTSNEANL (MTP) or MSLTTPPAVARP (MSL) [301] into the HI loop of CAR binding-ablated fiber gene. In both studies, efficacy and selectivity were also assessed in mice and not limited to primary cells in vitro, as opposed to the above-mentioned examples of peptide-mediated adenoviral targeting. Combining the mutation in the CAR-binding region with an EC-targeted peptide insertion, however, was not sufficient to abrogate liver accumulation of adenoviral vectors upon systemic administration, despite mediating increased vector transduction of blood vessels [301]. However, the insertion of the “NGR” peptide motif enabled targeting of EC in areas of induced neovascularization [210]. Increased tumor-targeting via the vasculature was achieved in mice by genetic insertion of the EC-targeted RGD motif into the adenoviral fiber protein to ablate the heparan sulfate proteoglycan-binding domain [239]. Usage of the iRGD motif further increased tumor penetration and anti-tumor effects of oncolytic adenoviral vectors [240]. Lastly, another successful strategy of adenoviral retargeting is the pseudotyping of adenovirus serotype 5 (Ad5) fiber with that of serotype Ad19p, leading to reduced transduction of liver tissue presumably by a reduced affinity for blood coagulation factors [231]. Additional insertion of phage-selected peptides HTTHREP and HITSLLS yielded selective targeting to the renal vasculature in rats [231].

#### 4.3.2. Retroviral Targeting of ECs

Retroviruses represent the most commonly used viral vectors for stable transduction of cells in vitro or ex vivo. Several FDA-approved ex vivo gene therapy products, such as Strimvelis, Yeskarta and Kymriah [302], are based on retroviral gene transfer. The family *Retroviridae* comprises multiple members of enveloped positive-strand RNA viruses with a diameter of approx. 100–120 nm. Their genome encodes for at least three groups of structural proteins, the group-specific antigen (Gag), the polymerase (Pol) and the envelope (Env). Receptor-binding is mediated by the surface subunits of the Env protein, followed by cell entry either via fusion of the viral envelope with the cellular lipid bilayer or via endocytosis. After fusion of viral and cellular lipid membranes, the capsid is released into the cytoplasm, where it disassembles. The viral RNA genome is converted into DNA by viral reverse transcriptase and enters the nucleus, where it is being integrated into the host genome by viral integrase [303]. Replication-deficient retroviral vectors have a packaging capacity of up to 8 kb [304].

Although gammaretroviruses belong to the first viral vectors, that have been developed in the 1980s, their potential for selective gene transfer to ECs is limited. Gammaretroviral vectors with unmodified envelope proteins only show poor transduction efficacy for primary ECs [305,306,307], often requiring multiple subsequent viral exposures to yield sufficient transduction efficacy [306,307]. Pseudotyping the most commonly used gammaretroviral vector murine leukemia virus (MLV) with the vesicular-stomatitis-virus (VSV) glycoprotein G helped to improve gene transfer to HUVECs [308]. However, improved transduction of MLV via VSV glycoprotein G seems to be mediated by the ubiquitous LDL receptor [309]; therefore, it cannot be considered selective for ECs. Improved but presumably non-specific transduction of HSVECs was achieved by pseudotyping MLV vectors with the envelope protein of gibbon ape leukemia virus (GALV) [310]. Alternative gammaretrovirus variants, such as the neuropathogenic MLV TR1.3, which selectively infects and destroys brain capillary ECs [311], might provide platforms for highly efficient vectors in the future. Lentivirus, another member of the retrovirus family, might be better suited for endothelial gene transfer than gammaretroviruses [312,313]. HIV-1-based lentiviral vectors were used to efficiently transduce HUVECs and human coronary artery endothelial cells (HCAECs) in vitro. Upon infusion into rat carotid arteries in vivo, the EC transduction rate was approx. 5% [313]. By combining VSV-pseudotyped lentiviral vectors with the EC-specific Tie2 promoter, De Plama et al. were able to achieve highly selective transgene expression in angiogenic tumor ECs upon intravenous injection into tumor-bearing mice [241]. Pseudotyping lentiviral vectors with a modified Sindbis virus envelope harboring the Fc-binding domain of protein A is a very promising approach to couple lentiviral particles to specific antibodies, enabling selective targeting [314]. Based on this work, Pariente et al. tested various promoters in combination with pseudotyped lentiviral vectors containing Sindbis virus envelope targeted to CD146 [315]. Screened on a panel of cells in vitro, CD146-targeted pseudotyped lentivirus showed high efficacy and selectivity of transgene expression driven by the Tie2 promoter in HUVECs [315]. Another group developed a Sindbis virus-pseudotyped lentiviral vector carrying a VEGFR2-specific nanobody, which might be able to specifically target tumor vasculature [316]. A very similar lentiviral targeting strategy has been developed by Yang et al. based on the incorporation of a target-specific antibody and a so-called fusogenic protein into the lentiviral surface [317]. The fusogen consisted of modified envelope proteins lacking affinity for their cognate receptor, while the ability to mediate pH-dependent membrane fusion was retained. Specific targeting to CD20 was tested as proof of concept, yielding particles with tropism for human B cells [317]. To our knowledge, this strategy has not been implemented for in vivo EC targeting, so far, but it certainly represents a promising technique to improve EC transduction by lentiviral vectors in the future. Taken together, some of the surface-modified lentiviral vectors system seem to allow for efficient and specific gene expression in ECs—especially when combined with transcriptional targeting strategies. However, selective targeting of EC subpopulations has not been achieved, so far, using lentiviral vectors.

#### 4.3.3. Adeno-Associated Viral Targeting of ECs

Adeno-associated virus (AAV) is a small, non-enveloped, single-stranded DNA virus with an icosahedric capsid of 20 nm in diameter. The 4.7 kb genome encodes the replication proteins (Rep) and the structural virion proteins (Cap), as well as the assembly activating protein (AAP). The packaging capacity of recombinant AAV vectors ranges around 5 kb, similar to the size of the natural AAV genome [318]. Similar to adenovirus and retrovirus, AAV serves as frequently used gene therapy vector, with, currently, two approved clinical applications, namely, Zolgensma and Luxturna, against spinal muscular atrophy and Leber congenital amaurosis, respectively [319]. AAV tropism is broad, as there are numerous distinct AAV serotypes with affinity for different cell surface molecules. Primary cell attachment is mostly mediated by heparan sulfate proteoglycan [320], or multiple other glycans of the ECM. This is followed by binding to integrins [321] or growth factor receptors [322]. The cellular uptake of most AAV serotypes by receptor-mediated endocytosis further requires the presence of the transmembrane dyslexia-associated protein KIAA0319, also termed AAVR (for AAV receptor) [323]. Strategies to re-direct the tropism of AAV to alternative cellular molecules include the following: conjugation or genetic insertion of previously identified targeting ligands, such as phage-selected peptides or antibodies, into the AAV capsid; random shuffling of different AAV serotype capsids; introduction of specific or random mutations into the cap gene; isolation of random peptide ligands presented within the AAV capsid by screening AAV display peptide libraries [319].

Many AAV serotypes possess the capacity to transduce ECs, although the transduction efficacy for most serotypes is limited. A comparison of the AAV serotypes 1–5 showed substantially stronger in vitro transduction of aortic ECs (human and rat) by AAV1, compared to the other serotypes, presumably being brought about by binding to sialic acid [324]. Another study comparing AAV serotypes 1–9 on a diverse panel of target cells showed that the transduction efficacy of AAV6 is superior to that of AAV1, whereas AAV2 is the third most efficient serotype in terms of HUVEC transduction [325]. The comparably high transduction efficacy of AAV2 for ECs was confirmed in other studies on HUVECs [326,327], HSVECs [326] and murine ECs [328], as well as the microvascular ECs of mice [329] and humans [327]. Although ranging among the more efficient AAV serotypes for EC transduction, AAV2 is outperformed by adenoviral vectors upon infusion into rabbit jugular veins [330]. Adenoviral vectors based on Ad5, as well as VSV-pseudotyped lentiviral vectors, also provide higher transduction efficacy for HUVECs and HSVECs, compared to AAV (serotypes 2–6) [326]. In addition, EC transduction by unmodified AAV vectors is, by far, not selective. Most AAV serotypes show strong tropism for liver, heart or muscle tissue, since classic primary and secondary AAV receptors are expressed on a broad range of cells [331]. Therefore, exactly as for the other viral vector platforms, selective EC targeting by AAV requires modifications of the viral surface. By screening a phage library on HUVECs, White et al. isolated several HUVEC-enriched peptides. The most strongly enriched variants MSLTTPPAVARP and MTPFPTSNEANL were inserted into the receptor-binding region of the AAV2 capsid at amino acid position I-587. The resulting AAV2 vectors efficiently transduced HUVECs in vitro and showed enrichment in the vena cava upon intravenous administration in mice, compared to unmodified control vectors [242]. Insertion of the phage-selected EC-targeted SIGYPLP peptide into the same region of the AAV2 capsid abrogated HSPG binding and increased affinity for HUVECs and HSVECs [332], as it has also been descried for adenoviral vectors [299]. Phage display has also been applied in vivo with the purpose of identifying peptides for the subsequent insertion into AAV2 vector capsids. Chen et al. used a mouse model of the MPS VII lysosomal storage disorder to identify phage-peptides either homing to healthy or diseased cerebral vascular ECs [197]. Two epitopes were found to be most strongly enriched in cerebral ECs upon intravenous injection, namely, WPFYGTP (“PFG”) in diseased MPS VII mice and DSPAHPS (“PPS”) in healthy heterozygous mice. AAV2 vectors transferring the coding sequence of β-glucuronidase were successfully used to treat MPS VII mice after inserting the PFG peptide into the receptor binding domain at amino acid position 587 [197]. Targeting ligands can also be screened directly in the context of the AAV capsid [333,334]. Screening a random AAV2 display peptide library with peptide insertion at amino acid position R588 on HCAECs, Müller et al. identified the peptide NDVRAVS mediating strongly increased affinity of AAV2 for HCAECs and strongly reduced affinity of non-target HeLa cells [333]. A similar AAV2 display peptide library, developed in parallel, has recently been screened on human macrovascular ECs and pluripotent stem cell-derived endothelial progenitor cells, yielding the peptides VSSSTPR and NNPLPQR. One of the corresponding AAV vectors, “AAV-V_EC_” (displaying the VSSSTPR peptide), showed superior transduction of HUVECs and endothelial progenitor cells, compared to all other tested variants, including wildtype AAV2 and the previously identified AAV2-NDVRAVS [335]. To our knowledge, in vivo selectivity of the above-mentioned AAV library-based capsid mutants has not been investigated so far. Members of our group used the AAV2 display peptide library to screen for variants with highly selective EC tropism in vivo. One identified AAV capsid mutant displaying the peptide “ESGHGYF” at amino acid position R588 was shown to completely restrict vector tropism to pulmonary ECs upon intravenous injection in mice, outperforming many other AAV-based EC targeting approaches in terms of in vivo selectivity [213]. “AAV-BR1”, another AAV capsid mutant isolated from our AAV2 library which displays the peptide “NRGTEWD”, mediates homing to ECs of the central nervous system (brain, spinal cord and retina) [194]. AAV-BR1 has been applied in various studies to assess the role of brain or retina ECs in different physiological and pathophysiological settings [336,337,338,339,340,341,342,343] and it has successfully been used in mouse models to treat the neurological impairments of incontinentia pigmenti [194,195] and Sandhoff disease [196]. In our view, the above-mentioned examples demonstrate huge experimental and therapeutic potential of AAV display peptide libraries and phage display libraries alike and both techniques will likely yield targeting peptides for additional EC subpopulations in the future.

## 5. Conclusions

Decades after electron microscopy had first enabled scientist to catch a glimpse of EC diversity, novel biotechnological methods, such as microarrays and (single cell) RNAseq, have helped to finally grasp the tremendous heterogeneity of vascular ECs. Vascular ECs can be assorted into a multitude of subpopulations according to morphology and function, which is reflected by highly distinctive gene expression signatures. Selective endothelial targeting is a necessity to assess the role of the diverse EC subpopulations and to deepen our understanding of their functions and elucidate therapeutic potential. Various methods have been proven useful to enable EC targeting in general. However, selective targeting of distinct EC subpopulations in vivo is more difficult and robust solutions are scarce. Although some of the discussed surface molecules might serve as specific markers for distinct EC subpopulations, truly selective targeting often requires a unique pattern of multiple cell surface molecules, composed of one or more protein receptors, as well as specific glycan structures contributing to the glycocalyx. Additional processing of the targeting agent upon cell binding can further increase selectivity. To this end, viral vectors hold exceptional promise, as vector-mediated gene expression relies on a multitude of processes (cell attachment, cell internalization, intracellular trafficking, endosomal escape, nuclear entry and gene expression), all of which can contribute to the selectivity. After carefully reviewing the existing literature, we conclude that most of the reported EC targeting approaches focus on cell binding and, due to the limited number of truly unique surface molecules, not all of these approaches successfully restrict EC targeting to a single subpopulation. Many of the studies reporting on successful organ-specific EC targeting or selective targeting of disease-specific ECs utilize the power of peptide libraries, either displayed on bacteriophages or on other viruses, such as AAV. While the therapeutic potential of EC-targeted interventions is already being investigated in clinical studies, new targeting molecules will be identified for additional EC subpopulations and each of these molecules may help to further dissect EC diversity at a functional level.

## Figures and Tables

**Table 1:** Examples of selective EC targeting.

EC Population	Species	Platform	Targeting Moiety	Application	Ref.
Cardiac ECs	Mouse	Bacteriophage	CRPPR peptide	-	[192]
Cerebral ECs	Mouse	Bacteriophage	CAGALCY peptide	-	[193]
		AAV2	NRGTEWD „BR1“ peptide (R588 insertion)	*Nemo* gene therapy of incontinentia pigmentii	[194,195]
				*Hex A/B* gene therapy of Sandhoff disease	[196]
			Phage-selected DSPAHPS (“PPS”) peptide (I587 insertion)	-	[197]
	Rat	Bacteriophage	QPEHSST peptide	-	[198]
Cerebral ECs (ischemic)	Mouse	Antibody	PECAM-1 paratope	Urokinase-type plasminogen activator treatment of ischemic stroke	[199]
Cerebral EC junctions	Mouse	Bacteriophage	Peptides harboring the “FRW” morif		[200]
Cerebral ECs (MPSVII mucopolysaccharidosis)	Mouse	AAV2	Phage-selected WPFYGTP (“PFG”) peptide (I587 insertion)	β-glucuronidase gene therapy	[197]
Dermal ECs	Human	Bacteriophage	CHGGVGSGC peptide	-	[201]
Dermal ECs (inflamed)	Mouse	Ad vector	E-selectin paratope of antibody conjugated via PEG	-	[202]
High endothelial venule ECs (lymph nodes)	Mouse	Nanoparticle	PNAd paratope of MECA-79 monoclonal antibody	Improvement of heart allograft survival	[203]
	Sheep	Antibody		Amelioration of asthma	[204]
Inflamed ECs	Mouse, human	Nanobody	VCAM-1 paratope	Imaging of atherosclerotic lesions by SPECT in mice	[205]
	Mouse	Antibody	PLVAP paratope	Treatment of endotoxin-mediated inflammation in mice with SOD-coupled antibody	[206]
		Nanobody	VCAM-1 paratope	Imaging of atherosclerotic lesions by PET/MRI in mice	[207]
				Imaging of atherosclerotic lesions by ultrasound in mice	[208]
Ischemic muscle ECs	Mouse	Ad vector	Targeted gene expression by PPE1-3x promoter	HIF-1α gene therapy	[209]
Neovascular ECs	Mouse	Ad5 vector	Phage-selected “NGR” peptide motif	-	[210]
Pulmonary ECs	Mouse	Bacteriophage	CGFECVRQCPERC (“GFE-1”) peptide targeting membrane dipeptidase	-	[211,212]
		AAV2	AAV-selected peptide ESGHGYF (588 insertion)	-	[213]
	Rat	Bacteriophage	VNTANST peptide	-	[198]
		Ad vector	ACE paratope of bi-specific antibody	BMPRII gene therapy in two rat models	[214,215]
				eNOS gene therapy of stroke-prone hypertension	[216]
			ACE paratope of bi-specific antibody and targeted gene expression by flt-1 promoter	-	[217]
		Antibody	ACE-paratope	Catalse treatment of lungs before transplantation	[218,219]
Fibrotic pulmonary ECs	Mouse	Antibody	PLVAP paratope	Treatment of pulmonary fibrosis with prostaglandin-coupled antibody	[220]
Ischemic pulmonary ECs	Mouse	Antibody	PECAM-1 paratope	Catalse treatment of acute lung injury	[221,222]
				Thrombomodulin treatment of acute lung injury	[223]
				Urokinase-type plasminogen activator treatment of pulmonary embolism	[224,225]
	Rat	Antibody	PECAM-1 paratope	Catalse treatment of lungs before transplantation	[226]
	Pig	Antibody	PECAM-1 paratope	Catalse treatment of lungs before transplantation	[227]
Prostate ECs	Human	Bacteriophage	IL-11Rα-binding CGRRAGGSC peptide	Treatment of metastatic prostate cancer in patients	[201,228]
Renal ECs	Mouse	Bacteriophage	CLPVASC peptide	-	[229]
	Mouse/rat	Bacteriophage	PKNGSDP peptide	-	[230]
			DSHKDLK peptide	-	[230]
	Rat	Ad19p pseudotyped Ad5 vector	Phage-selected peptides HTTHREP and HITSLLS	-	[231]
Tumor ECs	Mouse	Bacteriophage	α integrin-binding peptide harboring the RGD motif	-	[232,233]
			α integrin/Neuropilin1-binding peptide harboring iRGD motif	Imaging with iRGD-coated iron oxide nanoworms and tumor treatment with iRGD-coated abraxane	[234]
				Lymphocyte infiltration in a xenograft mouse model of gastric cancer	[235]
				Improved efficacy of the anti-cancer membrane-active peptide HPRP-A1	[236]
		AAVP vector	α integrin-binding peptide harboring the RGD motif	Transgene deliver to tumor EC	[237,238]
		Ad5 vector		-	[239]
				Oncolytic gene therapy	[240]
		VSC-pseudotyped lentivirus	Targeted gene expression by Tie2 promoter	-	[241]
*Vena cava* ECs	Mouse	AAV2	Phage-selected peptides MSLTTPPAVARP and MTPFPTSNEANL (587 insertion)	-	[242]
White fat ECs	Mouse	Bacteriophage	CKGGRAKDC peptide	Ablation of adipose tissue in obese mice by apoptosis-inducing KLAKLAK peptide	[243]

## Data Availability

Not applicable.

## Abbreviations

AAV	adeno-associated virus
ACE	angiotensin converting enzyme
AMD	age-related macular degeneration
BBB	blood–brain barrier
bFGF	basic fibroblast growth factor
BMAEC(s)	bone marrow arterial endothelial cell(s)
BMSEC(s)	bone marrow sinusoidal endothelial cell(s)
cREC(s)	cortical renal endothelial cell(s)
EC	endothelial cell(s)
eNOS	endothelial nitric oxide synthase
gCap(s)	general capillary cell(s)
gREC(s)	glomeruli renal endothelial cell(s)
GVB	gut vascular barrier
HAEC(s)	human aortic endothelial cell(s)
HCAEC(s)	human coronary artery endothelial cell(s)
HEV(s)	high endothelial venule(s)
HIF	hypoxia-induced factor
HSVEC(s)	human saphenous vein endothelial cell(s)
HUVEC(s)	human umbilical vein endothelial cell(s)
MAdCAM	mucosal vascular addressin cell adhesion molecule
MLV	murine leukemia virus
mREC(s)	medulla renal endothelial cell(s)
NO	nitric oxide
PAEC(s)	porcine aortic endothelial cell(s)
PECAM-1	platelet endothelial cell adhesion molecule 1
PEG	polyethylene glycol
PVLAP	plasmalemmal vesicle-associated protein
REC(s)	renal endothelial cell(s)
VCAM1	vascular cell adhesion molecule 1
VEGF(R)	vascular endothelial growth factor (receptor)
VSV	vesicular stromatitis virus
vWF	von Willebrand Factor
